# Failed Retrieval of an Inferior Vena Cava Filter

**DOI:** 10.7759/cureus.100620

**Published:** 2026-01-02

**Authors:** Robert B Murrell, Mitchell Fisher, Christopher Stewart, George Ladas, Samuel Groot, Austin Wagner, Suporn Sukpraprut-Braaten

**Affiliations:** 1 General Surgery, St. Mary's Hospital, Blue Springs, USA; 2 Medicine, Kansas City University of Medicine and Biosciences, Joplin, USA; 3 Vascular Surgery, Midwest Aortic and Vascular Institute, Kansas City, USA; 4 Graduate Medical Education, Kansas City University, Kansas City, USA; 5 Graduate Medical Education, Unity Health, Searcy, USA

**Keywords:** anticoagulant therapy, deep vein thrombosis (dvt), inferior vena cava filter retrieval, ivc filter retrieval, pulmonary embolism (pe)

## Abstract

Inferior vena cava (IVC) filters are devices used to lower the risk of critical pulmonary embolisms in patients with current deep vein thrombosis (DVT) or a high risk of developing such. These devices are used especially when there are contraindications to anticoagulants or when conservative therapy has failed. Typically, IVC filters are temporary and can be retrieved upon improvement in the patient's clotting status.

This case presents a 75-year-old female whose IVC filter retrieval failed. Once cleared by both cardiology and orthopedics, the decision was made to retrieve the IVC filter. First attempts at retrieval proved unsuccessful as the IVC filter was tilted approximately 30 degrees. Several attempts were first made to retrieve the filter using the snare-and-sheath technique to grab the hook at the top of the filter. The clinicians then also attempted to use endoscopic graspers, followed by several different laparoscopic graspers to aid in more precise gripping of either the hook or the tines of the filter. However, the grip was insufficient when attempting to retract it into the sheath. An additional venogram was then taken, which demonstrated further tilting of the IVC filter. The hook of the IVC filter appeared to reside in the origin of the right renal vein. After multiple failed attempts, a decision was made to leave the filter in place to avoid potential complications.

Retrieving IVC filters can present with difficulty. The development of advanced retrieval techniques, aside from the snare-and-sheath standard method, has helped increase the removal success rates, and complication rates remain low. Clinicians should exercise caution when IVC filters are difficult to retrieve, as this can lead to severe complications. Despite utilizing these techniques in complex scenarios, further study is needed to define and standardize the removal steps.

## Introduction

Deep vein thrombosis (DVT) is a common disorder of the venous circulatory system, typically characterized by thrombus formation in the deep veins of the calf, but it can occur elsewhere in the body [1,2]. It has an incidence of 1.6 in every 1,000 people annually [1]. The feared complication of DVT is progression into a pulmonary embolism (PE), an obstructive disorder causing blockage of any of the pulmonary arteries. This eventually can lead to respiratory failure and death in more severe cases [3]. To avoid this life-threatening condition, preventative treatment is essential and can be in various forms. These can include, but are not limited to, pharmacologic anticoagulation, compression stockings, or placement of an inferior vena cava (IVC) filter, among others. 

Inferior vena cava (IVC) filters have been in use since the 1960's and approximately 65,000 of these filters are placed each year in the United States [4,5]. IVC filters are commonly indicated as a treatment plan to prevent large pulmonary embolism in patients with venous thromboembolism who also have an absolute contraindication to anticoagulation therapy or who have had a progression to a deep vein thrombosis despite adequate anticoagulation [6]. 

Once the risk of a thrombus traveling to the lungs is acceptably low or if the patient can begin anticoagulation medication, retrievable IVC filters can be removed relatively safely [7,8]. Once the decision is made to retrieve an IVC filter, the standard technique utilized is the snare-and-sheath method. This method involves inserting a sheath into the right internal jugular vein. A small loop snare is then inserted through the sheath and is used to wrap around the IVC filter's hook that is located at the superior end of the filter. Ideally, the loop snare can grasp onto the hook and pull the filter device up into the sheath, where the sheath can then be retracted along with the IVC filter simultaneously. Despite a high success rate with the standard snare-and-sheath technique, several different advanced techniques have been developed in cases where the standard technique fails to retrieve the filter, which will be discussed later [9]. We report a case in which a retrievable IVC filter could not be removed despite using standard and several advanced techniques. Failure to retrieve these devices is a rare occurrence, and failure using multiple techniques is even more so. This case report is essential to the field of surgery as it contributes practical evidence to an understudied area of IVC filter retrieval. Further research needs to be conducted to standardize the steps for removal in these difficult scenarios.

## Case presentation

A 75-year-old female presented to the clinic in January 2024 for removal of her retrievable Denali™ IVC filter. She was previously diagnosed with a deep vein thrombosis in the right lower extremity after an orthopedic procedure nine months prior, and required subsequent inferior vena cava filter placement. Following placement of the IVC filter, she was started on rivaroxaban, a direct oral anticoagulant. She was eventually cleared by both cardiology and orthopedics to discontinue the rivaroxaban since she was treated for an adequate period of time following her provoked DVT. At this point, she no longer needed the IVC filter, so the decision was made to retrieve it. The figure below depicts an example of what the placement of the filter typically would look like.

**Figure 1:**
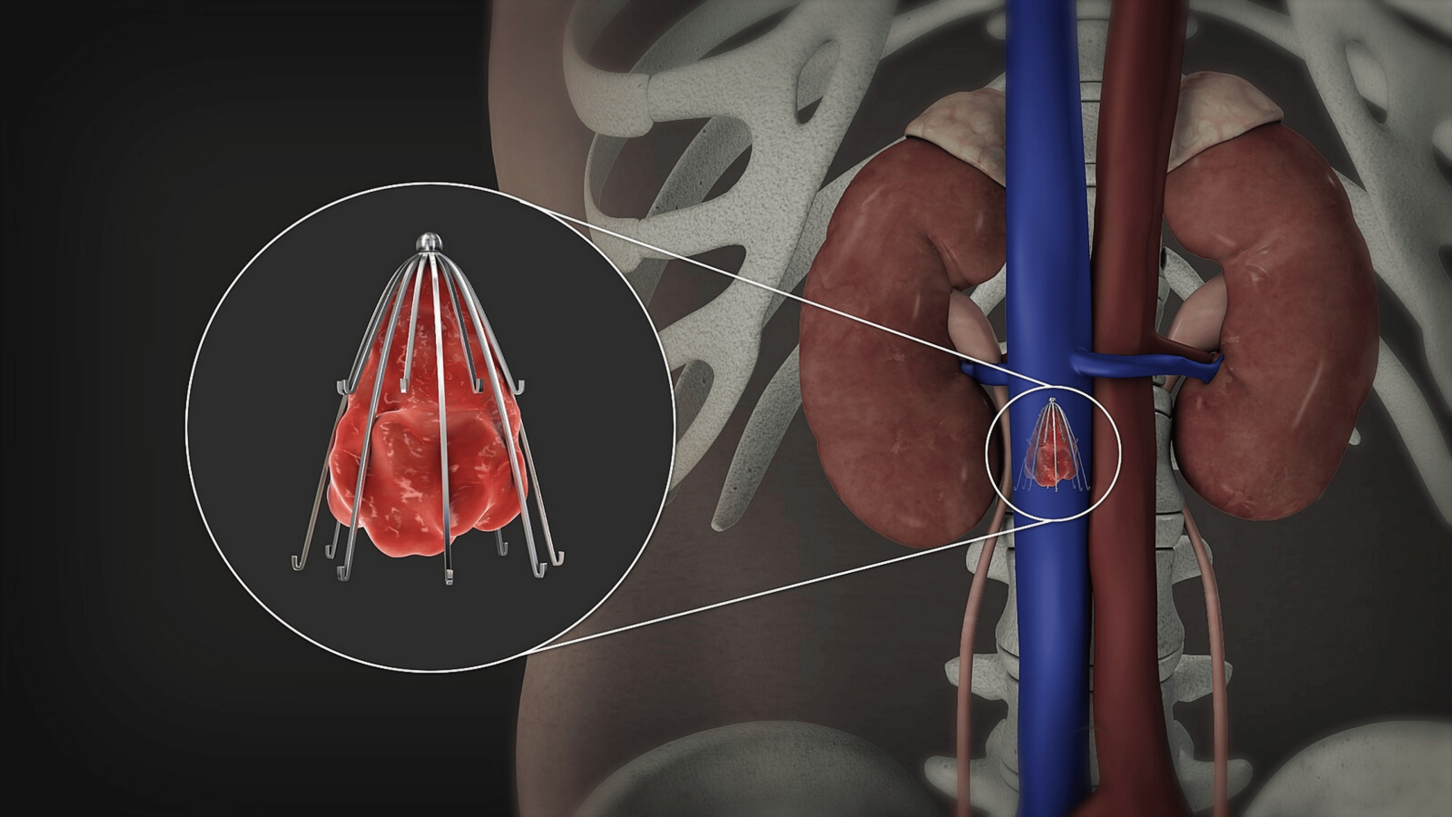
Inferior vena cava (IVC) filter reference image Reproduced from "3D Medical Animation Inferior Vena Filter", by Scientific Animations, https://www.scientificanimations.com, licensed under CC BY-SA 4.0.

The patient underwent an attempted retrieval of her IVC filter in an outpatient endovascular suite; however, the procedure was unsuccessful, due in part to angulation of the filter. Review of images demonstrated that the filter was appropriately placed at the index operation. A decision was then made to attempt IVC filter retrieval in the hospital's hybrid operating room. The right internal jugular vein was accessed with ultrasound guidance. A 6 French short sheath was then placed into the vein. A flush catheter was then introduced to eventually obtain our initial venogram.

**Figure 2:**
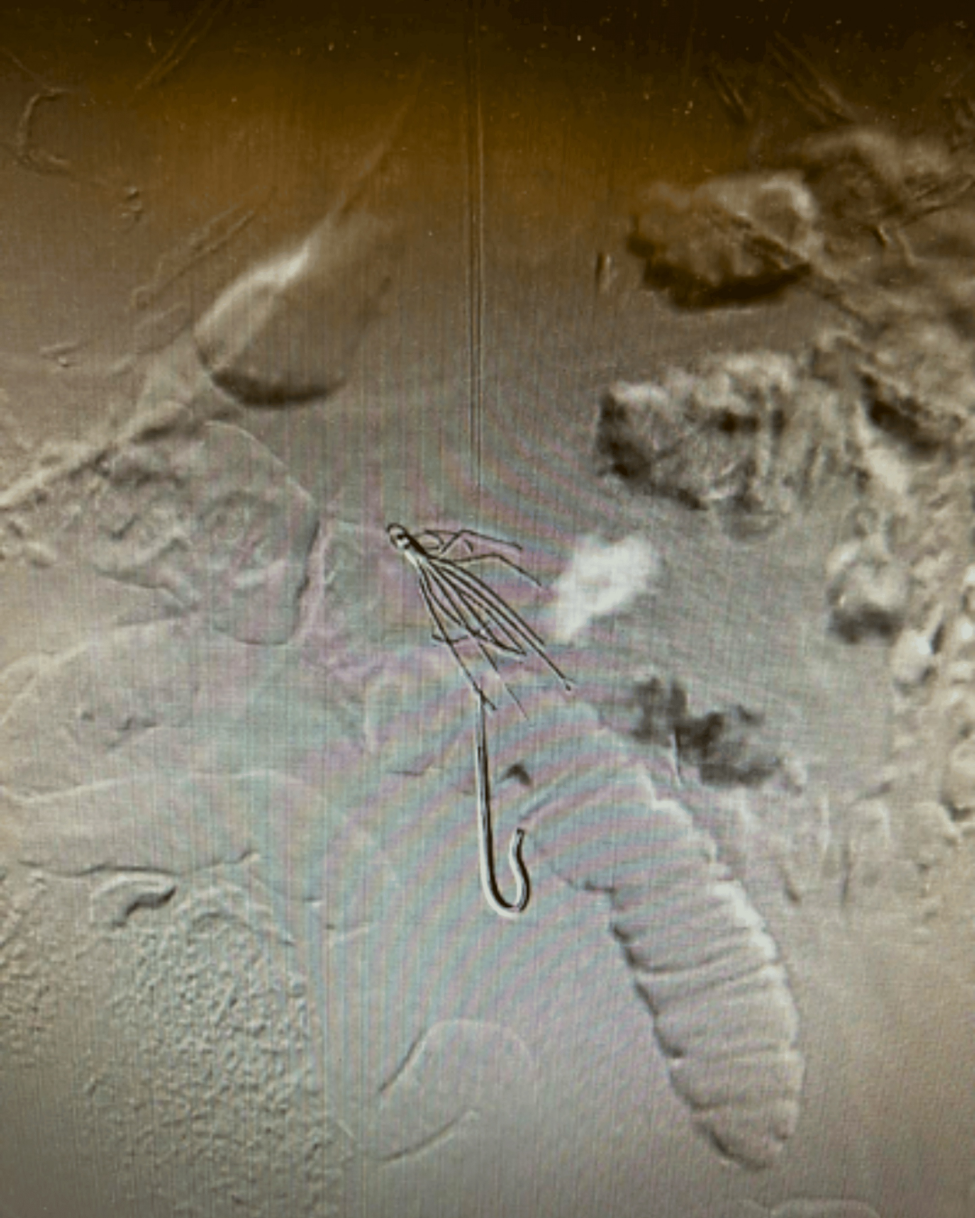
Initial fluoroscopic image demonstrating the IVC filter tilted at an angle IVC - inferior vena cava

**Figure 3:**
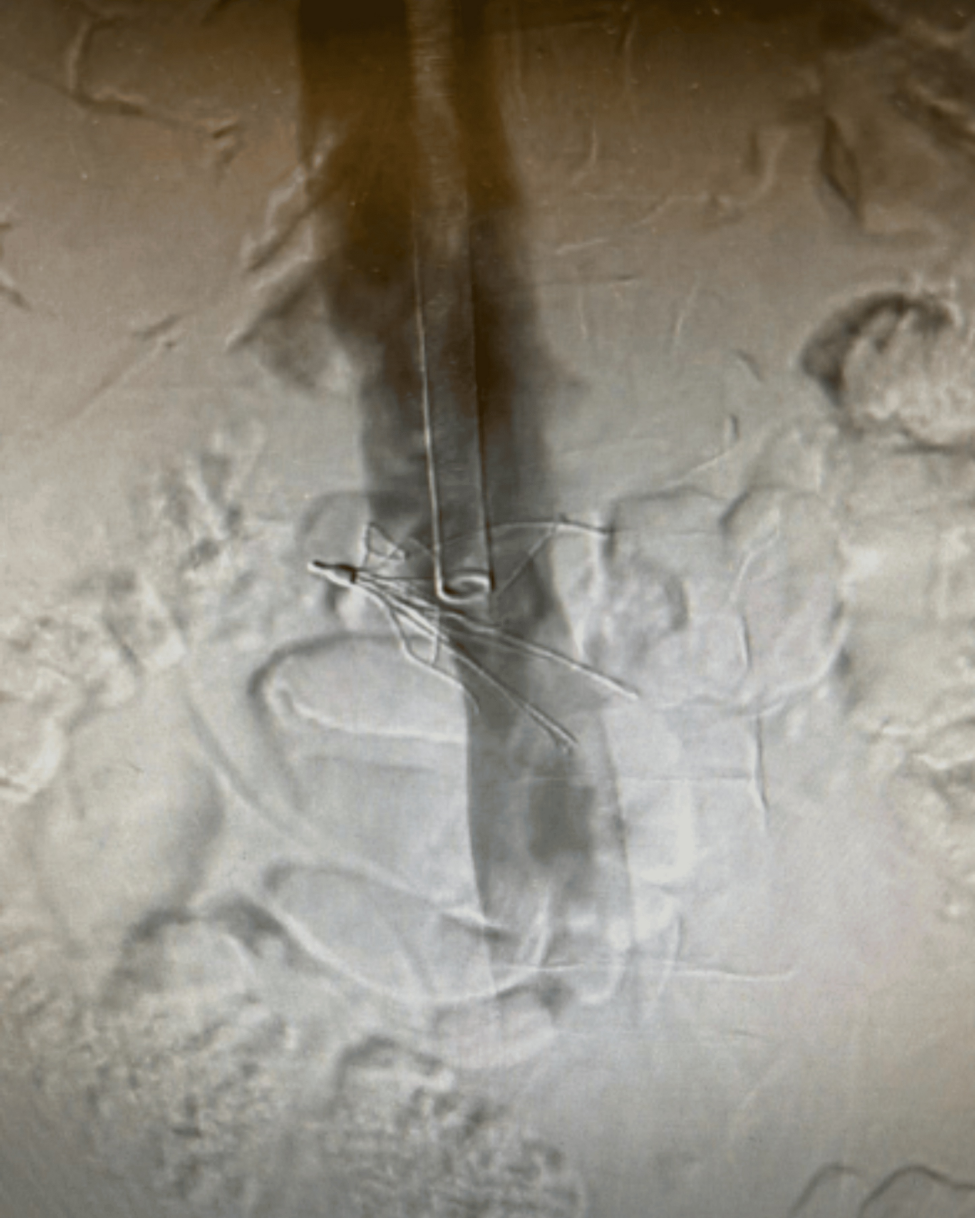
Final venogram image demonstrating further angulation of the IVC filter, likely in the right renal vein origin IVC - inferior vena cava

As shown in the above figure, the filter appeared to be tilted approximately 30 degrees. The sheath was then upsized to a 24 French sheath that was large enough for the removal of the IVC filter. Several attempts were first made to retrieve the filter using a snare-and-sheath technique to grab the hook at the top of the filter. The clinicians then attempted to use endoscopic graspers for more precise gripping of either the hook or the tines on the filter. The endoscopic graspers were not strong enough, so bariatric laparoscopic graspers were used. Several different laparoscopic graspers were utilized, which provided a stronger grip on the filter. However, due to the angle of the filter, the grip was insufficient when attempting to retract it into the sheath. An additional venogram was then taken, which demonstrated further tilting of the IVC filter, with the hook of the IVC filter residing in the right renal vein. After the multiple failed attempts, a decision was made to leave the filter in place to avoid potential complications. The image is shown below in Figure 3. The patient then followed up in our clinic a few weeks after the procedure, as well as at six and 12 months after. She did not have any complications related to the IVC filter and was educated on the risks associated with retained IVC filters. The patient was encouraged to follow up with our office as needed if she developed any new symptoms or complications.

## Discussion

Prior to retrievable IVC filters, permanent IVC filters were routinely used. IVC filters have been shown to be successful at preventing pulmonary embolism, yet appear to increase the risk of subsequent deep vein thrombosis in patients when compared to a control population who received no IVC filter [10]. When an IVC filter is no longer indicated, it is now common practice for IVC filters to be removed when the risk is reduced, or the patient can start anticoagulants. The standard technique for retrieval of an IVC filter is the snare-and-sheath technique, which is successful in 80-90% of cases [9]. This case presents an example where endoscopic and laparoscopic graspers were used as an advanced retrieval method, which still proved unsuccessful. 

Failure using the standard technique can usually be attributed to either filter tilt or adherence of the filter struts to the caval wall [11]. In one study, the failed retrieval rate appears to be close to 15% [4]. Another contributing factor to the difficulty of IVC filter retrieval is a prolonged dwell time [12]. Other variables of interest include filter types, retrieval indications, and retrieval methods [11]. In cases where retrieval is unsuccessful, advanced techniques can be utilized to increase the success of removal. Several different advanced techniques that have been described include: centering techniques, use of endo-bronchial forceps, use of bariatric laparoscopic graspers, coaxial double-sheath dissection, and laser-assisted double sheath dissection. Advanced techniques are becoming increasingly utilized; however, their safety and efficacy have not been closely studied [11]. A systematic review published in 2023 aimed to investigate the safety of complex IVC filter retrieval. Major complications occurred in 2.8% of patients during or after retrieval attempts with no reported patient mortality. Major complications localized to the IVC included thrombosis, pseudo-aneurysm, extravasation, dissection, non-occlusive stenosis, and retained filter strut within the IVC. Non-localized complications included retroperitoneal hemorrhage, access site complications, migration of the filter struts, and PE. Complication rate was not significantly associated with a specific filter type. Lastly, complications associated with a retained retrievable IVC filter are similar to those of permanent IVC filters: recurrent DVT, IVC thrombosis, filter fracture, and filter migration.

Despite the development of advanced techniques, there is limited information regarding the success rates of the various techniques used. This case report is useful because it outlines advanced techniques that have been described for removing an IVC filter that has already failed the standard. Furthermore, it demonstrates that there is a lack of data to support using one advanced technique over the other in terms of successful filter retrieval. Further research needs to be carried out for each advanced technique to determine the safety and efficacy of these procedures. This could prove useful in developing a standardized, step-wise approach to difficult IVC filter retrievals. Additionally, further research could help establish uniform international guidelines with an emphasis on reduced complication and failure rates for IVC filter retrieval. 

## Conclusions

This case report highlights that retrieval of IVC filters can be challenging, prompting clinicians to develop advanced techniques beyond the snare-and-sheath standard. The development of advanced techniques has helped increase the success of removing both retrievable and permanent IVC filters. Despite the high success rates of these techniques and their utilization in more complex scenarios, the complication rate remains low. Clinicians should exercise caution in scenarios where an IVC filter is difficult to retrieve, as it can lead to serious intraoperative complications. This case report is essential to the literature to better define procedural limits in these retrievals and assist in developing new evidence-based protocols driven toward patient safety when removal is difficult.

## References

[1] Waheed SM, Kudaravalli P, Hotwagner DT (2023). Deep Vein Thrombosis. NIH.

[2] Vyas V, Sankari A, Goyal A (2024). Acute Pulmonary Embolism. NIH.

[3] Neuhaus A, Bentz RR, Weg JG (1978). Pulmonary embolism in respiratory failure. Chest.

[4] Rodriguez AK, Goel A, Gorantla VR (2024). Complications associated with inferior vena cava filter retrieval: a systematic review. Cureus.

[5] Quencer KB, Smith TA, Deipolyi A, Mojibian H, Ayyagari R, Latich I, Ali R (2020). Procedural complications of inferior vena cava filter retrieval, an illustrated review. CVIR Endovasc.

[6] Bajda J, Park AN, Raj A, Raj R, Gorantla VR (2023). Inferior vena cava filters and complications: a systematic review. Cureus.

[7] Merritt T, Powell C, Hansmann J (2022). Safety and effectiveness of advanced retrieval techniques for inferior vena cava filters compared with standard retrieval techniques: a systematic review of the literature and meta-analysis. J Vasc Interv Radiol.

[8] DeYoung E, Minocha J (2016). Inferior vena cava filters: guidelines, best practice, and expanding indications. Semin Intervent Radiol.

[9] Ahmed O, Kim YJ, Patel MV, Tullius TG Jr, Navuluri R, Funaki B, Van Ha T (2020). A single-institutional comparative analysis of advanced versus standard snare removal of inferior vena cava filters. J Vasc Interv Radiol.

[10] Bikdeli B, Chatterjee S, Desai NR (2017). Inferior vena cava filters to prevent pulmonary embolism: systematic review and meta-analysis. J Am Coll Cardiol.

[11] Kethidi N, Barsoum K, Shukla PA, Kumar A (2023). Inferior vena cava filter retrievals using advanced techniques: a systematic review. Diagn Interv Radiol.

[12] Rosenthal D, Wellons ED, Hancock SM, Burkett AB (2007). Retrievability of the Günther Tulip vena cava filter after dwell times longer than 180 days in patients with multiple trauma. J Endovasc Ther.

